# Pulmonary Gas Embolism During Robot‐Assisted Partial Nephrectomy: Experiences, Current Trends, and Important Cautions in Japan

**DOI:** 10.1111/ases.70278

**Published:** 2026-03-25

**Authors:** Toshinori Nishikimi, Tomoyoshi Ohashi, Yujiro Hosono, Yuriko Nagasaka, Kaichi Sugihara, Hiroko Morikami, Masato Fukushima, Hiroshi Yamada

**Affiliations:** ^1^ Department of Urology Japanese Red Cross Aichi Medical Center, Nagoya Daini Hospital Nagoya Japan

**Keywords:** embolism, retroperitoneal space, robotic surgical procedures

## Abstract

In Japan, nine gas embolisms related to robot‐assisted partial nephrectomy (RAPN)—including fatal cases—have been reported, all with a retroperitoneal approach, predominantly at 15 mmHg intra‐abdominal pressure using gasketless cannulas. At our facility, a gas embolism occurred during retroperitoneal RAPN for an 18 × 14‐mm left renal cell carcinoma. During resection, intra‐abdominal pressure was maintained at 15 mmHg with positive end‐expiratory pressure deactivated. Seven minutes later, percutaneous oxygen saturation fell to 89%, accompanied by a decrease in end‐tidal carbon dioxide, leading to a diagnosis of pulmonary gas embolism. Postoperative plain computed tomography revealed a mirror image of CO_2_ from the inferior vena cava to the left femoral vein. The retroperitoneal approach, high intra‐abdominal pressure, and use of gasketless cannulas are considered risk factors for gas embolism. Prevention requires understanding these factors and setting appropriate intra‐abdominal pressures.

## 1 Introduction

Robot‐assisted partial nephrectomy (RAPN) has been covered by health insurance in Japan since April 2016, leading to an increase in procedures driven by benefits such as reduced ischemia time enabled by the advanced operability of robot systems. Reports on gas embolism related to RAPN are limited worldwide; however, in Japan, nine such cases—including fatal ones—have been reported, all with a retroperitoneal approach, predominantly at 15 mmHg intra‐abdominal pressure using gasketless cannulas. A case of a gas embolism occurred during RAPN at our facility. We have conducted a review of Japanese studies and evaluated associated risks.

## 2 Case Presentation

### 2.1 Patient

A 76‐year‐old male.

### 2.2 Chief Complaint

Detected left renal tumor on computed tomography (CT).

### 2.3 Past Medical History

Cholecystitis, right‐sided empyema.

### 2.4 Present Illness

**FIGURE 1:**
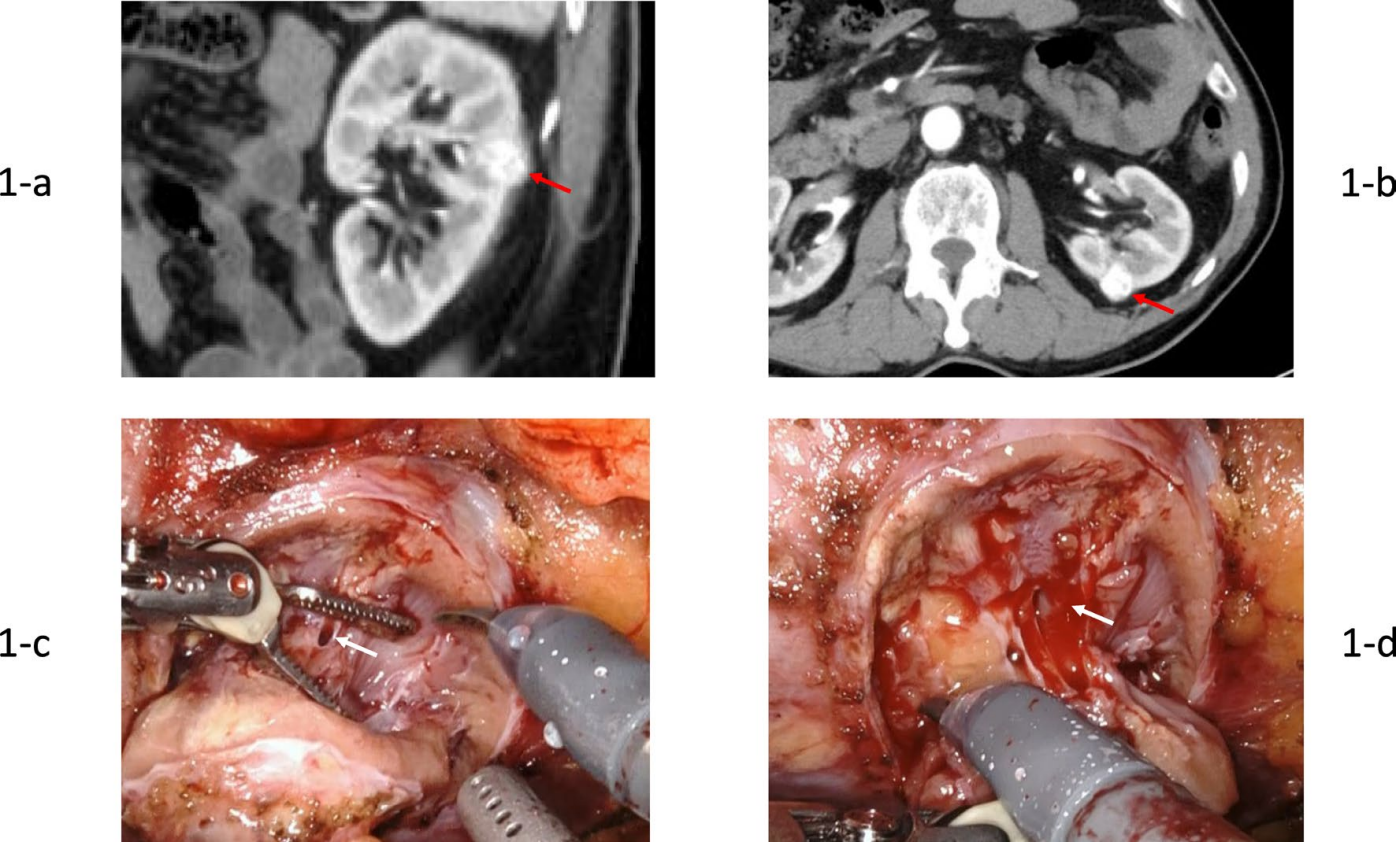
(a and b) Abdominal contrast‐enhanced CT. An 18 × 14 mm tumor was observed on the dorsal aspect of the left kidney, with marked contrast enhancement in the arterial phase (RENAL nephrometry score 6p). (c and d) Intraoperative findings. A small vein (1–2 mm) was opened approximately 4 min after initiating partial nephrectomy. Arrow indicates the opened vein.

While hospitalized for cholecystitis, a left renal mass was identified via CT imaging (Figure 1a,b), prompting referral. After 6 months, RAPN was performed.

### 2.5 Findings on Admission

Height, 160 cm; weight, 59 kg; body mass index, 23.05.

### 2.6 Surgical Procedure

**FIGURE 2:**
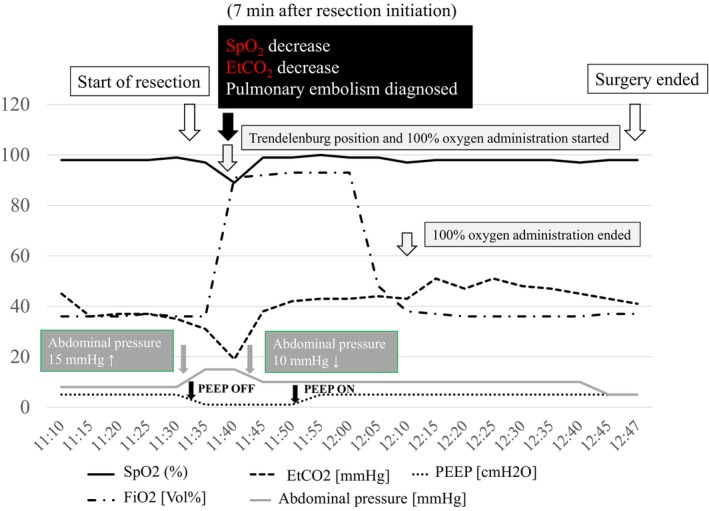
Seven minutes after initiating resection, the decreases in SpO_2_ and EtCO_2_ allowed for the diagnosis of a pulmonary gas embolism. The patient was immediately placed in a Trendelenburg position, and pure oxygen was administered. Additionally, the intra‐abdominal pressure was lowered to 10 mmHg and PEEP was discontinued. SpO_2_ and EtCO_2_ improved, and the surgery was concluded with oxygenation remaining stable.

The da Vinci Xi surgical robot (Intuitive Surgical) was used with the patient in the right lateral decubitus position. Six ports were used for RAPN via the retroperitoneal approach. The AirSeal Intelligent Flow System provided insufflation throughout the procedure. During tumor resection, intra‐abdominal pressure was maintained at 15 mmHg, and positive end‐expiratory pressure (PEEP) was disabled. Complete arterial clamping was established without venous clamping. Non‐resection phases utilized an intra‐abdominal pressure of 8 mmHg. Approximately 4 min after initiating resection, a small vein (1–2 mm) was opened (Figure 1c,d), and 7 min post‐initiation, percutaneous oxygen saturation decreased to 89% with systolic blood pressure falling to 70 mmHg. Decreased end‐tidal carbon dioxide partial pressure and increased arterial CO_2_ partial pressure indicated pulmonary gas embolism (Figure 2).

### 2.7 Immediate Interventions

The patient was placed in the Trendelenburg position, and intra‐abdominal pressure decreased to 10 mmHg at 9 min post‐resection initiation. The opened vein was closed using a single layer of inner sutures using the Sliding‐Clip method with 3–0 Vloc. The arterial clamp was released 15 min post‐initiation. Parenchymal outer suturing was performed with 2–0 Vloc, and hemostasis was confirmed before the surgery was concluded.

### 2.8 Operating Metrics

Total operating time, 180 min; console time, 120 min; warm ischemia duration, 15 min; minimal blood loss reported.

### 2.9 Postoperative Imaging

**FIGURE 3:**
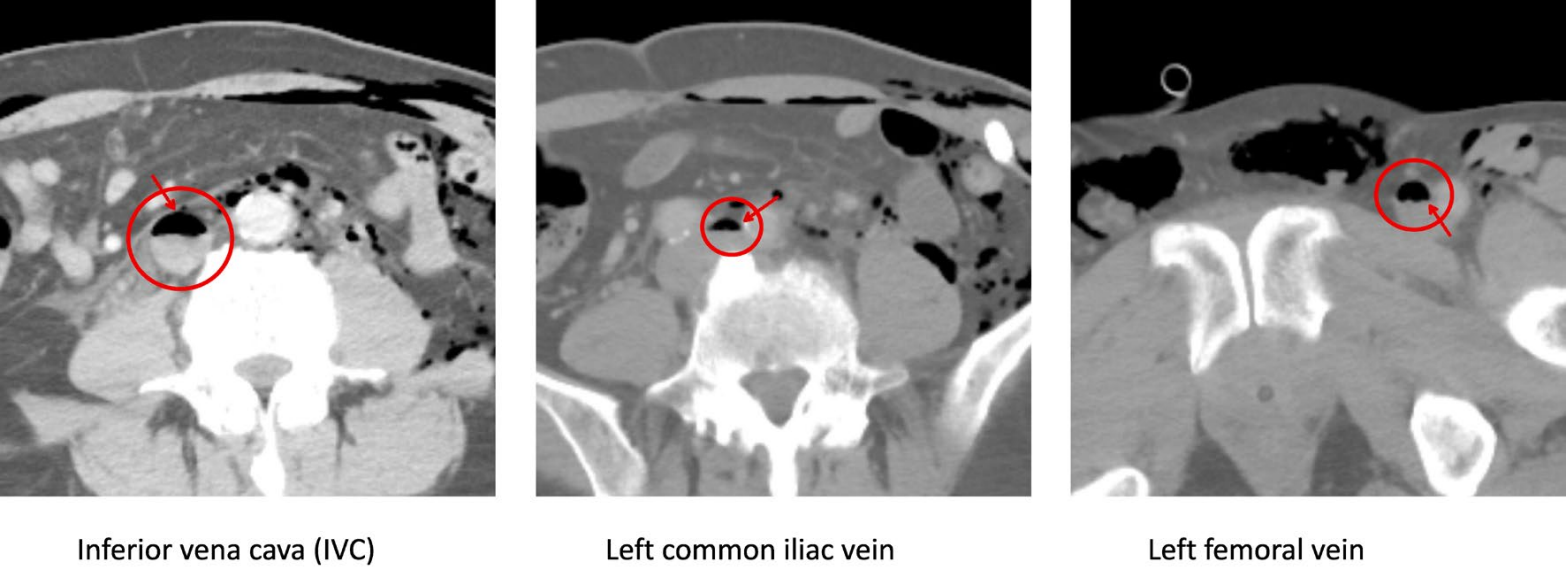
Postoperative CT findings. A mirror image of CO_2_ extending from the inferior vena cava (IVC) to the left femoral vein was observed.

Immediate non‐contrast chest and abdominal CT demonstrated no gas within the right atrium; however, a mirror image of CO_2_ extension from the inferior vena cava to the left femoral vein was observed (Figure 3).

### 2.10 Postoperative Course

Postoperatively, the patient was kept in the Trendelenburg position and maintained stable oxygenation. The following day, a follow‐up CT scan confirmed that the gas in the vein had resolved. No sequelae or complications occurred.

## 3 Gas Embolism in RAPN: Current Status in Japan

Since Gettman et al. [1] documented the first RAPN procedure for small renal cell carcinoma in 2004, and Shiraki et al. [2] followed by Japan's first case in 2011, various facilities have adopted this technique. In Japan, seven publications describe a total of nine cases of gas embolism associated with RAPN (Table 1) [3, 4, 5, 6, 7, 8, 9]. Although one report did not specify the surgical approach, all cases were performed using a retroperitoneal method. Intra‐abdominal pressure during resection was 15 mmHg in all but one case, which used 10 mmHg. The AirSeal Intelligent Flow System was used for insufflation in all nine cases.

**TABLE 1:** Case reports of gas embolism in RAPN in Japan.

Publication year	Author	Age	Sex	Affected side	Approach	cT stage	RENAL score	Intra‐abdominal pressure during resection	Gasketless cannula system use	Intracardiac right‐to‐left shunt (confirmed)	Cerebral infarction (presence/absence)
2020	Nishikimi et al.	76	Male	Left	Retroperitoneal	cT1a (18 mm)	6p	15 mmHg	Yes		No
2022	Nakagawa et al.	77	Female	Left	Retroperitoneal	cT1a (20 mm)	8p	15 mmHg	Yes		No
2023	Uchida et al.	76	Male	Right	Retroperitoneal	cT1b (45 mm)	10p	15 mmHg	Yes	Yes (TEE)	No
2023	Tomozane et al.	56	Male	Right	Unknown	Unknown	Unknown	15 mmHg	Yes	Yes (TEE)	Yes
2024	Inokuchi et al.	64	Male	Right	Retroperitoneal	cT1a (size unknown)	7p	15 mmHg	Yes		No?
2024	Inokuchi et al.	47	Male	Left	Retroperitoneal	cT1a (size unknown)	9a	15 mmHg	Yes		No?
2024	Inokuchi et al.	77	Male	Right	Retroperitoneal	cT1b (size unknown)	Unknown	15 mmHg	Yes	Yes (autopsy)	Yes
2025	Shiga et al.	83	Male	Right	Retroperitoneal	cT1a (38 mm)	7p	15 mmHg	Yes	Yes (TEE)	Yes
2025	Mori et al.	71	Male	Right	Retroperitoneal	cT1b (41 mm)	Unknown	10 mmHg	Yes	Yes (TEE)	Yes

Abbreviation: TEE, transesophageal echocardiography.

## 4 Discussion

We identified the retroperitoneal approach, an intra‐abdominal pressure of 15 mmHg, and use of the AirSeal Intelligent Flow System as potential risk factors for gas embolism during RAPN.

RAPN techniques are generally categorized into transperitoneal and retroperitoneal approaches, paralleling conventional laparoscopic procedures. Notably, the retroperitoneal approach has been recognized as offering more direct access for dorsal tumors [10, 11]. In the current case study, this method was selected owing to the tumor's dorsal location. However, the retroperitoneal approach presents a limited operative field, and combining high intra‐abdominal pressure with the AirSeal Intelligent Flow System may increase the risk of gas embolism.

Increasing intra‐abdominal pressure to 15 mmHg during resection is acknowledged as a potential risk factor for gas embolism, particularly in the confined space typical of the retroperitoneal approach. Notably, while maintaining intra‐abdominal pressure at 15 mmHg appears to increase risk, a case of gas embolism has been documented at 10 mmHg [9]. The presented case of gas embolism represented the 61st RAPN procedure performed at our hospital. Before this case, intra‐abdominal pressure routinely increased from 10 mmHg at the onset of surgery to 15 mmHg during resection, with PEEP deactivated. However, following this incident of gas embolism, procedural adjustments were made: intra‐abdominal pressure was maintained at 8 mmHg and increased to 10 mmHg during resection, with PEEP deactivation or further pressure elevation reserved for situations such as significant bleeding. Since implementing these changes, over 100 RAPN procedures have been performed without subsequent gas embolism. Thus, addressing each case individually rather than uniformly adjusting intra‐abdominal pressure and PEEP can help reduce the incidence of gas embolism based on the amount of bleeding during resection.

Regarding insufflation devices, all nine Japanese cases of gas embolism involved the use of the AirSeal Intelligent Flow System. This device offers stable intra‐abdominal pressure and optimal visualization via intermittent smoke evacuation and efficient insufflation [12]. While advantageous, early venous opening in a restricted retroperitoneal space may still predispose to gas embolism owing to characteristics of the system. Furthermore, gasketless cannula systems like AirSeal Intelligent Flow System, unlike conventional gasketed designs, utilize room air to sustain abdominal insufflation, thereby increasing the risk of non‐absorbable gas embolism [13]. At higher aspiration rates, CO_2_ is increasingly replaced by non‐medical room air, increasing the risk of poorly absorbed gases, such as oxygen and nitrogen. Specifically, at aspiration rates > 25 L/min, the standard gasketed cannula system desufflates, while the gasketless system continues to entrain air to maintain insufflation: 64% room air at 30 L/min aspiration, 71% at 40 L/min aspiration, 77% at 50 L/min aspiration, and 84% at 60 L/min aspiration. Of the nine Japanese cases, one fatality was attributed to cerebral gas embolism owing to non‐absorbable gas [7].

Gas embolism during laparoscopic surgery typically causes venous embolism, but cerebral embolism may occur if gas enters the left heart, resulting in arterial embolism. Contributing factors include intracardiac shunts, such as atrial septal defects and patent foramen ovale (PFO). Catheter interventions requiring atrial septal puncture can create iatrogenic atrial septal defects (iASDs), which persist for varying durations post‐procedure: iASD occurs in approximately 90% of patients immediately after catheter ablation, decreasing to 5% after 12 months; after left atrial appendage occlusion, iASD occurs in approximately 80%, 50%, and 10%–20% at 1, 6, and 12 months, respectively [14, 15]. Consequently, patients with a history of such interventions may be at increased risk of cerebral gas embolism from right‐to‐left shunts. However, asymptomatic PFOs, present in approximately 15%–20% of healthy individuals [16], pose a risk. Of the nine reported cases of gas embolism in Japan, four developed cerebral gas embolism [6, 7, 8, 9], with right‐to‐left shunt confirmed in three cases by autopsy or transesophageal echocardiography.

We deactivated PEEP during tumor resection to reduce venous bleeding, although this remains controversial. While lowering PEEP may decrease venous oozing, it can promote atelectasis and impair oxygenation. Conversely, increasing PEEP may help prevent gas entry through open veins but could raise right atrial pressure in patients with an intracardiac right‐to‐left shunt, potentially facilitating gas migration into the left heart system [17].

Among the nine Japanese cases, six occurred on the right and three on the left, suggesting right‐sided predominance (Table 1). This may reflect anatomical differences, as the right renal vein is shorter and drains directly into the inferior vena cava; however, additional cases are needed to confirm this trend. Temporary renal vein clamping during resection may be preventive, but venous dissection is more challenging in the retroperitoneal than in the transperitoneal approach, limiting its routine use.

Regarding tumor anatomical characteristics, seven of nine cases reported a RENAL Nephrometry score (mean, 6.7: 1 high‐, 5 intermediate‐, and 1 low‐risk), indicating few high‐risk tumors. Among four cases with cerebral gas embolism, one lacked tumor data; of the remaining three, two were cT1b and one was 38 mm (cT1a), all relatively large (Table 1). The case with embolism at 10 mmHg was cT1b (41 mm). These findings suggest that larger tumor diameter may increase the risk of gas embolism. In summary, the retroperitoneal approach, intra‐abdominal pressure of 15 mmHg, and use of the AirSeal Intelligent Flow System have been identified as risk factors in reported Japanese cases of gas embolism associated with RAPN. This case underscores the necessity of understanding procedural nuances and equipment characteristics, as well as establishing appropriate intra‐abdominal pressure settings, for effective prevention of gas embolism associated with retroperitoneal RAPN.

## Author Contributions

**Toshinori Nishikimi:** methodology, investigation, writing – original draft, Writing – review and editing. **Tomoyoshi Ohashi:** writing – review and editing. **Yujiro Hosono:** writing – review and editing. **Yuriko Nagasaka:** writing – review and editing. **Kaichi Sugihara:** writing – review and editing. **Hiroko Morikami:** writing – review and editing. **Masato Fukushima:** writing – review and editing. **Hiroshi Yamada:** writing – review and editing. All authors have read and approved the final version of the manuscript.

## Ethics Statement

The protocol for this research project was approved by the appropriate Ethics Committee at the institution (registration No. 001219). Informed consent was obtained from the patient.

## Conflicts of Interest

The authors declare no conflicts of interest.

## Supporting information


**Video S1:** The auxiliary surgical video: Six ports were used for RAPN via the retroperitoneal approach. The AirSeal Intelligent Flow System provided insufflation throughout the procedure.

## Data Availability

The data that support the findings of this study are available on request from the corresponding author. The data are not publicly available due to privacy or ethical restrictions.
